# Comparative Rehabilitation Benefits of Water-Based Versus Land-Based Exercise in Patients with Chronic Obstructive Pulmonary Disease: A Systematic Review and Meta-Analysis

**DOI:** 10.3390/life16020207

**Published:** 2026-01-27

**Authors:** Weiping Du, Jianhua Zhou, Aiping Chi

**Affiliations:** 1 School of Physical Education, Ningxia Normal University, Guyuan 756099, China; 82015006@nxnu.edu.cn (W.D.); 17866544603@163.com (J.Z.); 2 School of Physical Education, Shaanxi Normal University, Xi’an 710119, China

**Keywords:** chronic obstructive pulmonary disease, water-based exercise, land-based exercise, pulmonary rehabilitation, meta-analysis

## Abstract

Patients with chronic obstructive pulmonary disease (COPD) commonly experience impaired lung function, reduced exercise tolerance, and respiratory muscle weakness. Owing to the unique properties of the aquatic environment, water-based exercise may provide rehabilitation benefits that differ from those of traditional land-based exercise. **Objective**: This systematic review and meta-analysis aimed to compare the effects of water-based versus land-based exercise on lung function, exercise capacity, and respiratory muscle function in patients with COPD, thereby providing evidence to inform the optimization of pulmonary rehabilitation exercise modalities. **Methods**: PubMed, Web of Science, CNKI, and other databases were systematically searched to identify randomized controlled trials comparing water-based and land-based exercise interventions in adults with COPD. Primary outcomes included lung function (FEV_1_% predicted and FEV_1_/FVC), exercise capacity (six-minute walk distance, 6MWD), respiratory muscle strength (maximal inspiratory pressure (MIP]) and maximal expiratory pressure (MEP). Meta-analyses were performed using Stata 17.0. **Results:** A total of 14 RCTs were included. Meta-analysis showed that, compared with land-based exercise, water-based exercise significantly improved FEV_1_% predicted (WMD = 3.33, 95% CI: 0.02–6.64) and FEV_1_/FVC (WMD = 4.00, 95% CI: 1.27–6.73). Regarding exercise capacity, water-based exercise significantly increased 6MWD (WMD = 47.81 m, 95% CI: 20.19–75.44), with more pronounced improvements observed in short-term interventions (≤8 weeks). Respiratory muscle function analyses demonstrated significant improvements in MIP (WMD = 14.22 cmH_2_O, 95% CI: 7.75–20.69) and MEP (WMD = 14.40 cmH_2_O, 95% CI: 4.92–23.89). **Conclusions:** Compared with land-based exercise, water-based exercise demonstrates consistent advantages in improving exercise capacity and respiratory muscle function in patients with COPD and shows additional benefits for lung function indices. Therefore, water-based exercise may serve as a valuable adjunct to land-based training within pulmonary rehabilitation programs.

## 1. Introduction

Chronic obstructive pulmonary disease (COPD) is a common chronic respiratory disorder characterized by persistent airflow limitation, with its prevalence and global disease burden continuing to increase worldwide [1,2,3]. In addition to progressive declines in lung function, patients with COPD frequently experience marked reductions in exercise tolerance, respiratory muscle weakness, and limitations in activities of daily living. These impairments substantially compromise quality of life and are associated with an increased risk of acute exacerbations, hospitalization, and mortality [4,5]. Although pharmacological therapy plays an important role in symptom relief and exacerbation prevention, its effects on improving exercise capacity and functional status are limited. Consequently, non-pharmacological interventions, particularly pulmonary rehabilitation centered on exercise training, have become an integral component of comprehensive COPD management [5].

Pulmonary rehabilitation is consistently recommended by multiple international guidelines as a standard therapeutic strategy for patients with stable COPD, with structured exercise training recognized as the core element for improving exercise tolerance, alleviating dyspnea, and enhancing overall functional status [4,5,6]. Conventional pulmonary rehabilitation programs predominantly rely on land-based exercise modalities, including walking, cycling, aerobic training, and resistance exercise, which have been shown to improve exercise performance and functional outcomes to some extent [4]. However, in clinical practice, many patients with COPD present with comorbid lower-limb muscle weakness, joint degeneration, obesity, or moderate-to-severe dyspnea. As a result, some patients exhibit poor tolerance to land-based exercise and encounter difficulties in maintaining moderate- to high-intensity training over the long term, thereby limiting the feasibility and adherence of exercise interventions [7,8,9].

Against this background, water-based exercise (also referred to as aquatic exercise) has increasingly attracted attention as an alternative or complementary training modality [10,11]. The aquatic environment possesses distinct physical properties, including buoyancy, water resistance, and hydrostatic pressure, which may confer physiological advantages that differ from those of land-based exercise for patients with COPD [12]. Buoyancy reduces body-weight loading and joint stress, facilitating safer exercise participation among individuals with limited mobility or musculoskeletal constraints [13]. Water resistance provides uniform and adjustable loading even at relatively low movement velocities, thereby promoting combined improvements in muscular strength and endurance [12,13]. In addition, hydrostatic pressure exerted on the thorax and abdomen increases respiratory workload and may induce training effects on both inspiratory and expiratory muscles, potentially enhancing respiratory muscle function [14]. Collectively, these characteristics suggest that water-based exercise may offer unique advantages in improving exercise capacity, respiratory muscle strength, and overall functional status in patients with COPD [15].

From a functional-outcome perspective, the disease burden of COPD is primarily reflected in ventilatory impairment, reduced overall exercise capacity, and respiratory muscle dysfunction [4]. Among lung function indices, the percentage of predicted forced expiratory volume in one second (FEV_1_% predicted) serves as a key indicator of airflow limitation and disease severity, whereas the ratio of forced expiratory volume in one second to forced vital capacity (FEV_1_/FVC) is widely used to characterize airway obstruction and to evaluate treatment efficacy [4]. Exercise capacity is commonly assessed using the six-minute walk distance (6MWD), which reflects functional performance at intensities close to daily activities and demonstrates high clinical sensitivity and prognostic value in pulmonary rehabilitation settings [16,17]. Furthermore, respiratory muscle function plays a critical role in the development and progression of COPD. Maximal inspiratory pressure (MIP) primarily reflects the strength of the diaphragm and inspiratory muscle groups, whereas maximal expiratory pressure (MEP) is used to evaluate expiratory muscle function [18,19]. These parameters are commonly employed to assess the comprehensive effects of different exercise interventions on functional outcomes in COPD.

In recent years, several randomized controlled trials (RCTs) have investigated the effects of water-based exercise on lung function, exercise capacity, and quality of life in patients with COPD. Some studies have reported beneficial effects of water-based exercise on 6MWD, respiratory muscle strength, and exercise adherence [20]. However, existing studies exhibit substantial heterogeneity with respect to sample size, intervention modalities and training parameters, outcome selection, and follow-up duration, resulting in inconsistent findings across trials [10,20].

Moreover, although land-based exercise remains the conventional and widely implemented modality in pulmonary rehabilitation, systematic evidence directly comparing the relative effects of water-based exercise and land-based exercise in patients with COPD—particularly regarding key functional outcomes such as lung function, exercise capacity, and respiratory muscle function—remains limited. This lack of comprehensive evidence synthesis constrains clinicians’ ability to make evidence-based decisions when selecting optimal exercise modalities for different patient populations [10].

Therefore, the present study conducted a systematic review and meta-analysis to synthesize evidence from existing RCTs and to comprehensively compare the effects of water-based versus land-based exercise on lung function, exercise capacity, and respiratory muscle function in patients with COPD. The aim was to provide a more objective, robust, and clinically relevant evidence base to inform the optimization of exercise prescriptions within pulmonary rehabilitation programs.

Based on the unique biomechanical and respiratory physiological characteristics of the aquatic environment, together with preliminary findings from previous studies, we proposed the following hypotheses: (1) compared with land-based exercise, water-based exercise would lead to greater improvements in exercise capacity, as reflected by significant increases in 6MWD; (2) water-based exercise would result in superior enhancements in respiratory muscle function, as indicated by greater improvements in MIP and MEP; and (3) with respect to lung function, water-based exercise would produce comparable or slightly greater improvements than land-based exercise, as reflected by changes in FEV_1_% predicted and FEV_1_/FVC.

## 2. Materials and Methods

### 2.1. Study Design and Registration

This study was conducted as a systematic review and meta-analysis and was reported in accordance with the Preferred Reporting Items for Systematic Reviews and Meta-Analyses (PRISMA) statement [21]. The methodological procedures followed the Cochrane Handbook for Systematic Reviews of Interventions (Version 6) [22]. The study protocol was registered in the International Platform of Registered Systematic Review and Meta-analysis Protocols (INPLASY; registration number: INPLASY202610046). All key methodological components, including the research question, eligibility criteria, search strategy, and planned analyses, were defined prior to study selection and data extraction.

### 2.2. Search Strategy

In accordance with the PRISMA guidelines, a systematic literature search was performed to identify studies examining the effects of water-based versus land-based exercise interventions on patients with chronic obstructive pulmonary disease (COPD). The following electronic databases were searched from inception to October 2025: PubMed, Web of Science, China National Knowledge Infrastructure (CNKI), Wanfang Data, and VIP Database.

The search focused on the effects of water-based exercise (water-based or aquatic exercise) and land-based exercise on functional outcomes in patients with COPD, including lung function, exercise capacity, and respiratory muscle strength. Eligible studies were required to include adult patients with a confirmed diagnosis of COPD and to adopt a randomized controlled trial or controlled interventional design. The intervention had to involve at least one form of water-based exercise and include a comparator consisting of land-based exercise or conventional pulmonary rehabilitation.

A combination of Medical Subject Headings (MeSH) terms and free-text keywords was used. The main search terms included, but were not limited to: “chronic obstructive pulmonary disease” OR “COPD”; “aquatic exercise” OR “water-based exercise” OR “hydrotherapy”; and “land-based exercise” OR “exercise training” OR “pulmonary rehabilitation”. Boolean operators (“AND” and “OR”) were applied as appropriate. In addition, reference lists of all included studies were manually screened to identify potentially eligible articles that may have been missed during the electronic search.

All literature searches and study selection procedures were conducted independently by two reviewers. Any discrepancies were resolved through discussion or, when necessary, by consultation with a third reviewer. Inter-rater agreement for study selection was assessed using Cohen’s kappa coefficient. The level of agreement between the two independent reviewers was [κ = 0.89], indicating substantial agreement. A detailed summary of the search strategy is provided in Table 1.

### 2.3. Eligibility Criteria

Studies were eligible for inclusion if they met all of the following criteria: (1) Study design: Clinical interventional studies, including randomized controlled trials (RCTs). (2) Participants: Adult patients with a confirmed diagnosis of chronic obstructive pulmonary disease (COPD), with no restrictions on sex or disease stage. (3) Interventions: Interventions: The experimental group received water-based exercise interventions (also referred to as aquatic exercise), such as water-based aerobic exercise, breathing-focused exercise, aquatic rehabilitation programs, or water-based traditional exercise. The intervention duration and training frequency had to be clearly reported. (4) Comparators: The control group received land-based exercise, conventional pulmonary rehabilitation training, or usual care. (5) Outcomes: At least one of the following outcomes was reported: lung function indices (FEV_1_% predicted and/or FEV_1_/FVC), exercise capacity (six-minute walk distance, 6MWD), or respiratory muscle strength (maximal inspiratory pressure, MIP, and/or maximal expiratory pressure, MEP). (6) Data availability: Outcome data were required to be reported in a format suitable for meta-analysis, such as mean ± standard deviation (SD), mean ± standard error (SE), or other statistics convertible to means and SDs.

Studies were excluded if they met any of the following criteria: (1) review articles, systematic reviews, meta-analyses, case reports, conference abstracts, animal studies, or in vitro experiments; (2) studies enrolling non-COPD populations or participants with severe comorbid conditions (e.g., advanced cardiovascular disease, neurological disorders, or malignancies), unless results for COPD patients were reported separately; (3) interventions that did not include water-based exercise or studies in which the effects of water-based and land-based exercise could not be clearly distinguished; (4) studies that did not report the outcomes of interest or lacked extractable quantitative data, and for which the required data could not be obtained by contacting the authors; (5) duplicate publications reporting overlapping data, in which case only the study with the most complete information or the largest sample size was retained.

### 2.4. Data Extraction

Data extraction was independently performed by two reviewers, and the extracted data were cross-checked for accuracy. The following basic information was collected from each included study: first author, year of publication, sample size, participant characteristics (including age, sex, and disease severity), and detailed descriptions of the intervention and comparator.

Intervention-related data focused on exercise modality (water-based or land-based exercise), exercise intensity, training frequency, and total intervention duration. With respect to outcomes, data relevant to the present meta-analysis were extracted, including lung function indices (FEV_1_% predicted and FEV_1_/FVC), exercise capacity (six-minute walk distance, 6MWD), and respiratory muscle strength (maximal inspiratory pressure, MIP, and maximal expiratory pressure, MEP).

For continuous variables, changes from baseline to post-intervention (change scores) were preferentially extracted as means and standard deviations (or values that could be converted accordingly). Any disagreements during data extraction were resolved through discussion; if consensus could not be reached, a third reviewer was consulted.

Across all included studies, baseline disease severity was comparable between the water-based exercise and land-based exercise groups, as reflected by similar baseline lung function indices (e.g., FEV_1_% predicted and FEV_1_/FVC). Most participants were classified as having moderate to severe COPD. The general characteristics of the included studies are summarized in Table 2.

### 2.5. Outcomes

The primary outcomes of this study included indices of lung function, exercise capacity, and respiratory muscle strength, which were used to systematically evaluate the functional effects of water-based versus land-based exercise interventions in patients with COPD.

Lung function outcomes primarily included the percentage of predicted forced expiratory volume in one second (FEV_1_% predicted) and the ratio of forced expiratory volume in one second to forced vital capacity (FEV_1_/FVC). FEV_1_% predicted is a key indicator of the degree of airflow limitation and disease severity, whereas FEV_1_/FVC is widely used to characterize airway obstruction and has important clinical relevance in the diagnosis and evaluation of treatment efficacy in COPD.

Exercise capacity was assessed using the six-minute walk distance (6MWD). The 6MWD is a commonly used functional measure that reflects overall exercise tolerance and the ability to perform activities of daily living in patients with COPD. It is highly sensitive to changes induced by pulmonary rehabilitation interventions and has been widely applied in both clinical practice and research settings.

Respiratory muscle strength outcomes included maximal inspiratory pressure (MIP) and maximal expiratory pressure (MEP). MIP primarily reflects the functional status of the diaphragm and inspiratory muscle groups, whereas MEP reflects the strength of expiratory muscles. These indices are frequently used to evaluate the effects of respiratory muscle training and pulmonary rehabilitation interventions on respiratory muscle function.

The aforementioned outcomes are widely employed in pulmonary rehabilitation and exercise intervention studies in COPD and collectively capture patients’ responses to different exercise modalities across multiple dimensions, including airway function, exercise capacity, and respiratory muscle strength. Accordingly, they were selected as the core outcome variables for the present meta-analysis.

### 2.6. Risk of Bias Assessment

To systematically evaluate the methodological quality and potential risk of bias of the included studies, the Cochrane Risk of Bias 2 (RoB 2) tool was applied to assess the randomized controlled trials. This tool evaluates potential sources of bias that may affect the validity of intervention effects across five domains: bias arising from the randomization process, bias due to deviations from intended interventions, bias due to missing outcome data, bias in outcome measurement, and bias in the selection of the reported results.

Based on the signaling questions within each domain, the risk of bias was judged as low risk of bias, moderate risk of bias, or high risk of bias, and an overall risk-of-bias judgment was subsequently derived for each study. The risk-of-bias assessment focused on the primary outcomes of the present study, including FEV_1_% predicted, FEV_1_/FVC, six-minute walk distance (6MWD), maximal inspiratory pressure (MIP), and maximal expiratory pressure (MEP).

Risk-of-bias assessments were conducted independently by two reviewers. Any disagreements were resolved through discussion. Detailed results of the quality assessment are presented in Table 3.

### 2.7. Statistical Analysis

Statistical analyses were performed using Stata version 17.0 (StataCorp LLC, College Station, TX, USA) and Review Manager (RevMan) version 5.4 (The Cochrane Collaboration, London, UK). Meta-analyses were conducted using the metan and metareg commands in Stata. Effect sizes were expressed as weighted mean differences (WMDs) or standardized mean differences (SMDs), with corresponding 95% confidence intervals (CIs).

Statistical heterogeneity among studies was assessed using Cochran’s Q test and the I^2^ statistic. A fixed-effects model was applied when heterogeneity was low (I^2^ < 50% and *p* > 0.10); otherwise, a random-effects model based on the DerSimonian–Laird method was used. To examine the robustness of the pooled results, leave-one-out sensitivity analyses were performed using the metainf command to evaluate the influence of each individual study on the overall effect estimates.

Subgroup analyses were conducted for all outcomes according to intervention duration (>8 weeks vs. ≤8 weeks). Intervention duration was prespecified as a potential effect modifier based on evidence from previous pulmonary rehabilitation literature. The cut-off of 8 weeks was chosen a priori to ensure adequate numbers of studies and outcome data within each subgroup, while remaining consistent with commonly reported durations of pulmonary rehabilitation programs [10,15,23,27,32,34].

Publication bias was assessed for all outcomes using Begg’s rank correlation test and Egger’s regression test [35,36], in conjunction with visual inspection of funnel plot symmetry. A *p* value < 0.05 was considered indicative of potential publication bias. Meta-regression analyses were considered but not performed due to the limited number of studies available for each outcome and the inconsistent reporting of potential continuous covariates across included studies.

## 3. Results

### 3.1. Study Selection

**Figure 1:**
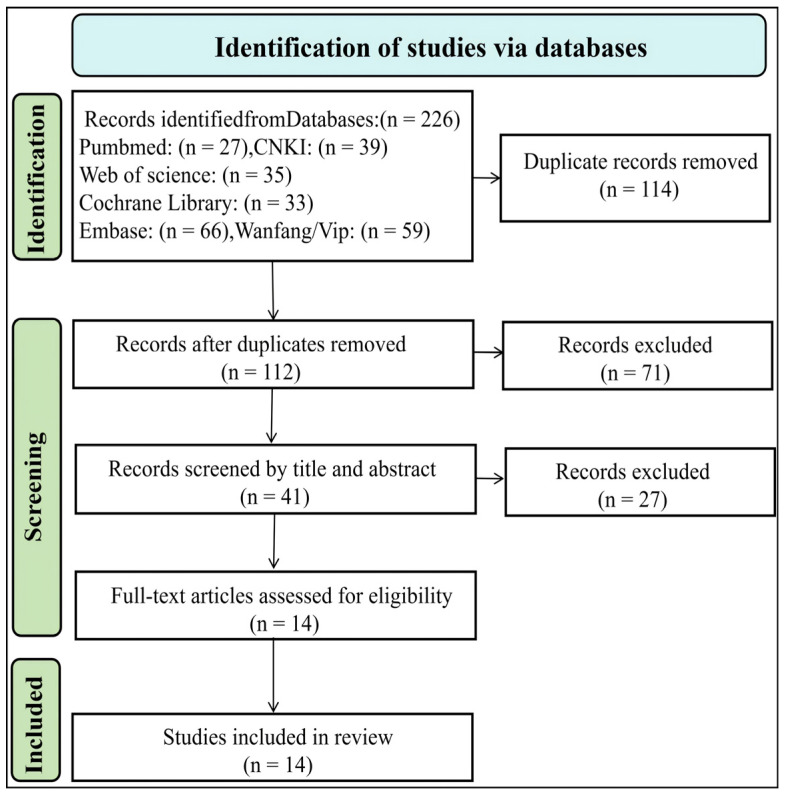
Flowchart of Literature Screening.

The literature selection process is illustrated in Figure 1. A total of 226 records were identified through the predefined search strategy. After removing 114 duplicate records, 112 studies remained for title and abstract screening. Of these, 71 records were excluded for failing to meet the eligibility criteria. Consequently, 41 full-text articles were assessed for eligibility. After full-text review, 27 articles were excluded based on the predefined inclusion and exclusion criteria. Ultimately, 14 studies were included in the final quantitative synthesis.

Among the 14 included studies, seven were judged to have an overall low risk of bias, reflecting clearly described randomization procedures, objective outcome measurements, and generally good data completeness. The remaining seven studies were assessed as having an overall moderate risk of bias, primarily due to the inherent difficulty of blinding participants and personnel in exercise intervention studies, partial missing outcome data, and the absence of clearly reported pre-registered protocols.

Overall, the risk of bias across the included studies was predominantly low to moderate. No clustering of studies with a systematically high risk of bias was observed, supporting the methodological robustness of the pooled estimates derived from the present meta-analysis.

### 3.2. Effects on Lung Function

#### 3.2.1. FEV_1_% Predicted

**Figure 2:**
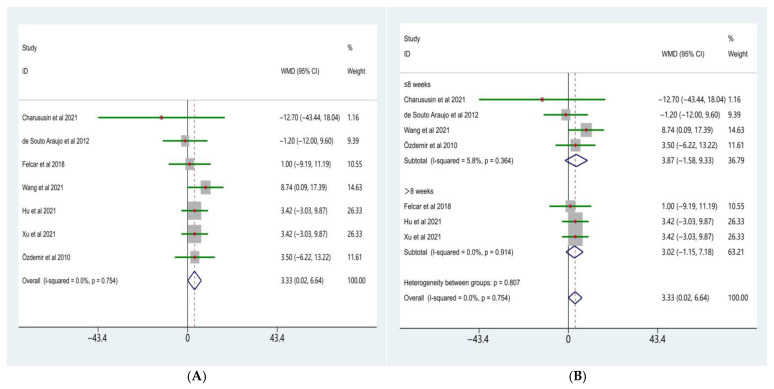
Forest plot of the meta-analysis for FEV1% predicted [15,23,24,26,29,30,32]. (**A**) Water-based exercise vs. land-based exercise on FEV1% predicted in patients with COPD; (**B**), Subgroup analysis based on intervention duration (≤8 weeks vs. >8 weeks) of the effect of water-based vs. land-based exercise on FEV1% predicted.

The meta-analysis of FEV_1_% predicted is presented in Figure 2A. Seven randomized controlled trials comparing the effects of water-based and land-based exercise on FEV_1_% predicted in patients with COPD were included. Heterogeneity testing indicated no significant between-study heterogeneity (χ^2^ = 3.42, df = 6, *p* = 0.754; I^2^ = 0.0%); therefore, a fixed-effects model was applied.

The pooled analysis demonstrated that, compared with land-based exercise, water-based exercise significantly improved FEV_1_% predicted in patients with COPD, with a weighted mean difference (WMD) of 3.33 (95% CI: 0.02–6.64). This difference reached statistical significance (Z = 1.97, *p* = 0.049).

To further explore the potential influence of intervention duration on treatment effects, subgroup analyses were performed according to intervention length (Figure 2B). The results showed no significant differences in the effects of water-based versus land-based exercise on FEV_1_% predicted between the different duration subgroups (test for subgroup differences, *p* = 0.807).

In studies with an intervention duration of ≤8 weeks, the pooled effect size was WMD = 3.87 (95% CI: −1.58 to 9.33; Z = 1.39, *p* = 0.164), with low heterogeneity across studies (I^2^ = 5.8%, *p* = 0.364). In studies with an intervention duration of >8 weeks, the pooled effect size was WMD = 3.02 (95% CI: −1.15 to 7.18; Z = 1.42, *p* = 0.156), and no significant heterogeneity was observed (I^2^ = 0.0%, *p* = 0.914).

#### 3.2.2. FEV_1_/FVC

**Figure 3:**
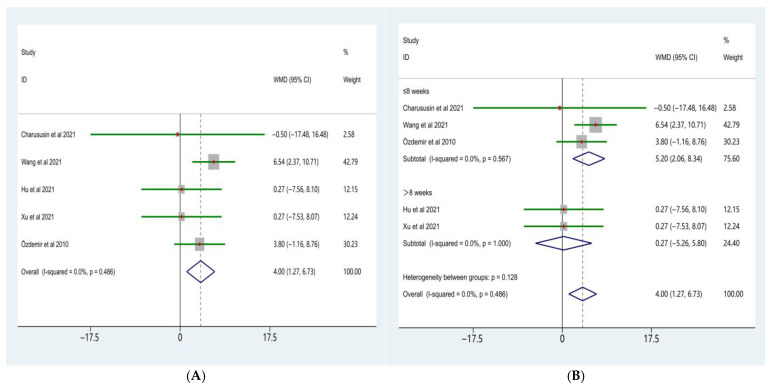
Forest plot of the meta-analysis for FEV1/FVC [23,26,29,30,32]. (**A**), Water-based exercise vs. land-based exercise on FEV1/FVC in patients with COPD; (**B**), Subgroup analysis based on intervention duration (≤8 weeks vs. >8 weeks) of the effect of water-based vs. land-based exercise on FEV1/FVC.

The meta-analysis of FEV_1_/FVC is shown in Figure 3A. Five randomized controlled trials comparing the effects of water-based and land-based exercise on FEV_1_/FVC in patients with COPD were included. Heterogeneity testing indicated no significant between-study heterogeneity (χ^2^ = 3.45, df = 4, *p* = 0.486; I^2^ = 0.0%); therefore, a fixed-effects model was applied.

The pooled analysis demonstrated that, compared with land-based exercise, water-based exercise significantly improved FEV_1_/FVC in patients with COPD, with a weighted mean difference (WMD) of 4.00 (95% CI: 1.27–6.73). This difference was statistically significant (Z = 2.87, *p* = 0.004).

Subgroup analyses were further conducted according to intervention duration (Figure 3B). The results showed that the effect of water-based exercise on FEV_1_/FVC did not differ significantly between subgroups with different intervention durations (test for subgroup differences, *p* = 0.128).

In studies with an intervention duration of ≤8 weeks, water-based exercise resulted in a significant improvement in FEV_1_/FVC compared with land-based exercise (WMD = 5.20, 95% CI: 2.06–8.34; Z = 3.25, *p* = 0.001), with no significant heterogeneity observed among studies (I^2^ = 0.0%, *p* = 0.567). In contrast, in studies with an intervention duration of >8 weeks, no statistically significant difference was observed between water-based and land-based exercise (WMD = 0.27, 95% CI: −5.26 to 5.80; Z = 0.10, *p* = 0.924), and heterogeneity remained negligible (I^2^ = 0.0%, *p* = 1.000).

Overall, the meta-analysis indicated that water-based exercise demonstrated significant advantages over land-based exercise in improving lung function indices, including FEV_1_% predicted and FEV_1_/FVC, in patients with COPD. However, these effects did not show statistically significant differences between subgroups stratified by intervention duration.

### 3.3. Effects on Exercise Capacity

**Figure 4:**
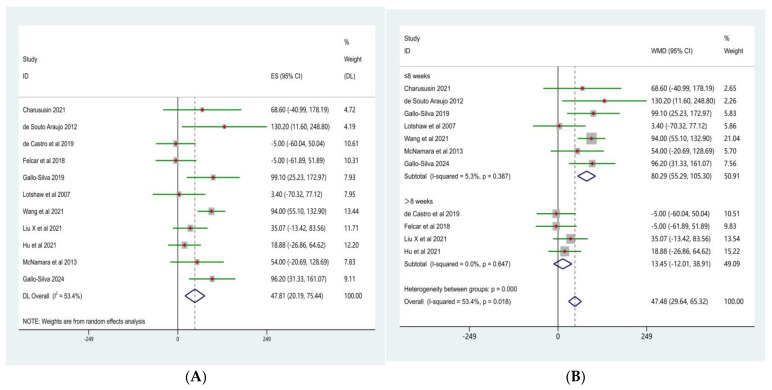
Forest plot of the meta-analysis for 6MWD [10,15,23,24,27,28,30,32,33,34]. (**A**), Water-based exercise vs. land-based exercise on 6MWD in patients with COPD; (**B**), Subgroup analysis based on intervention duration (≤8 weeks vs. >8 weeks) of the effect of water-based vs. land-based exercise on 6MWD.

The meta-analysis of the six-minute walk distance (6MWD) is presented in Figure 4A. Eleven randomized controlled trials comparing the effects of water-based and land-based exercise on 6MWD in patients with COPD were included. Heterogeneity testing revealed a moderate level of between-study heterogeneity (χ^2^ = 21.47, df = 10, *p* = 0.018; I^2^ = 53.4%); therefore, a random-effects model was applied.

The random-effects meta-analysis demonstrated that, compared with land-based exercise, water-based exercise significantly increased 6MWD in patients with COPD, with a pooled weighted mean difference (WMD) of 47.81 m (95% CI: 20.19–75.44). This difference was statistically significant (Z = 5.22, *p* < 0.001), indicating an overall advantage of water-based exercise in improving exercise capacity.

To further examine the potential influence of intervention duration on improvements in 6MWD, subgroup analyses were conducted according to intervention length (Figure 4B). The results showed a significant difference in the effects of water-based exercise on 6MWD between subgroups with different intervention durations (test for subgroup differences, *p* < 0.001).

In studies with an intervention duration of ≤8 weeks, water-based exercise resulted in a significant increase in 6MWD compared with land-based exercise (WMD = 80.29 m, 95% CI: 55.29–105.30; Z = 6.29, *p* < 0.001), with low between-study heterogeneity (I^2^ = 5.3%, *p* = 0.387). In contrast, in studies with an intervention duration of >8 weeks, the improvement in 6MWD associated with water-based exercise did not reach statistical significance (WMD = 13.45 m, 95% CI: −12.01 to 38.91; Z = 1.04, *p* = 0.301), and no significant heterogeneity was observed (I^2^ = 0.0%, *p* = 0.647).

Overall, the meta-analysis indicated that water-based exercise significantly improved exercise capacity compared with land-based exercise in patients with COPD. Intervention duration appeared to be an important moderating factor, with more pronounced improvements observed in short-term interventions (≤8 weeks).

### 3.4. Effects on Respiratory Muscle Function

#### 3.4.1. Maximal Inspiratory Pressure (MIP)

**Figure 5:**
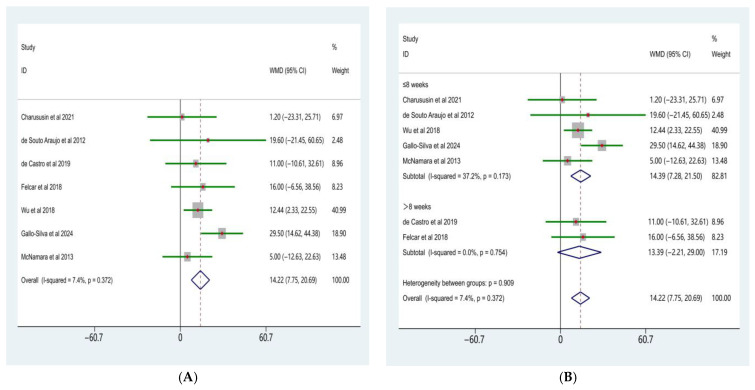
Forest plot of the meta-analysis for MIP [10,15,23,24,25,27,33]. (**A**), Water-based exercise vs. land-based exercise on MIP in patients with COPD; (**B**), Subgroup analysis based on intervention duration (≤8 weeks vs. >8 weeks) of the effect of water-based vs. land-based exercise on MIP.

The meta-analysis of maximal inspiratory pressure (MIP) is presented in Figure 5A. Seven randomized controlled trials comparing the effects of water-based and land-based exercise on inspiratory muscle strength in patients with COPD were included. Heterogeneity testing indicated a low level of between-study heterogeneity (χ^2^ = 6.48, df = 6, *p* = 0.372; I^2^ = 7.4%); therefore, a fixed-effects model was applied.

The pooled analysis demonstrated that, compared with land-based exercise, water-based exercise significantly increased maximal inspiratory pressure in patients with COPD, with a weighted mean difference (WMD) of 14.22 cmH_2_O (95% CI: 7.75–20.69). This difference was statistically significant (Z = 4.31, *p* < 0.001). Inspection of the forest plot showed that the effect estimates of most included studies were located to the right of the line of no effect, indicating a consistent positive effect of water-based exercise on inspiratory muscle strength.

Further subgroup analyses were conducted according to intervention duration (Figure 5B). The results indicated no significant differences in the effects of water-based versus land-based exercise on MIP between subgroups with different intervention durations (test for subgroup differences, *p* = 0.909).

In studies with an intervention duration of ≤8 weeks, the pooled effect size was WMD = 14.39 cmH_2_O (95% CI: 7.28–21.50; Z = 3.97, *p* < 0.001), with low-to-moderate heterogeneity across studies (I^2^ = 37.2%, *p* = 0.173). In studies with an intervention duration of >8 weeks, the pooled effect size was WMD = 13.39 cmH_2_O (95% CI: −2.21 to 29.00; Z = 1.68, *p* = 0.093), and no significant heterogeneity was observed (I^2^ = 0.0%, *p* = 0.754).

#### 3.4.2. Maximal Expiratory Pressure (MEP)

**Figure 6:**
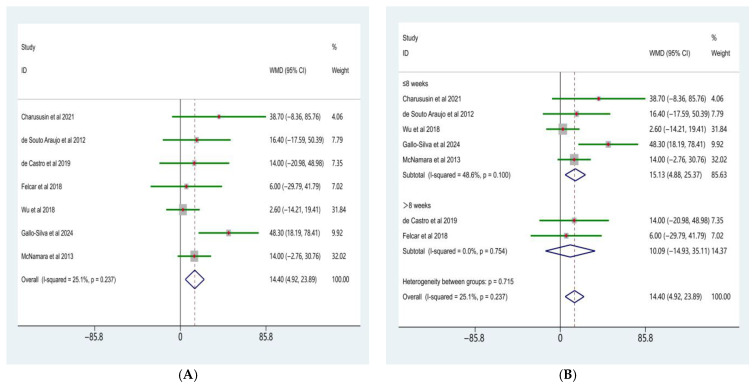
Forest plot of the meta-analysis for MEP [10,15,23,24,25,27,33]. (**A**), Water-based exercise vs. land-based exercise on MEP in patients with COPD; (**B**), Subgroup analysis based on intervention duration (≤8 weeks vs. >8 weeks) of the effect of water-based vs. land-based exercise on MEP.

The meta-analysis of maximal expiratory pressure (MEP) is presented in Figure 6A. Seven randomized controlled trials comparing the effects of water-based and land-based exercise on expiratory muscle strength in patients with COPD were included. Heterogeneity testing indicated a low level of between-study heterogeneity (χ^2^ = 8.02, df = 6, *p* = 0.237; I^2^ = 25.1%); therefore, a fixed-effects model was applied.

The pooled analysis showed that, compared with land-based exercise, water-based exercise significantly increased MEP in patients with COPD, with a weighted mean difference (WMD) of 14.40 cmH_2_O (95% CI: 4.92–23.89). This difference was statistically significant (Z = 3.02, *p* = 0.003).

In Figure 6B, Subgroup analyses based on intervention duration indicated no significant differences in the effects of water-based versus land-based exercise on MEP between subgroups with different intervention lengths (test for subgroup differences, *p* = 0.715).

In studies with an intervention duration of ≤8 weeks, the pooled effect size was WMD = 15.13 cmH_2_O (95% CI: 4.88–25.37; Z = 2.89, *p* = 0.004), with a moderate level of heterogeneity across studies (I^2^ = 48.6%, *p* = 0.100). In studies with an intervention duration of >8 weeks, the pooled effect size was WMD = 10.09 cmH_2_O (95% CI: −14.93 to 35.11; Z = 0.79, *p* = 0.429), and no significant heterogeneity was observed (I^2^ = 0.0%, *p* = 0.754).

Overall, the results suggest that water-based exercise confers an overall advantage over land-based exercise in improving expiratory muscle strength in patients with COPD, whereas the moderating effect of intervention duration on this outcome remains unclear.

### 3.5. Sensitivity Analysis

**Figure 7:**
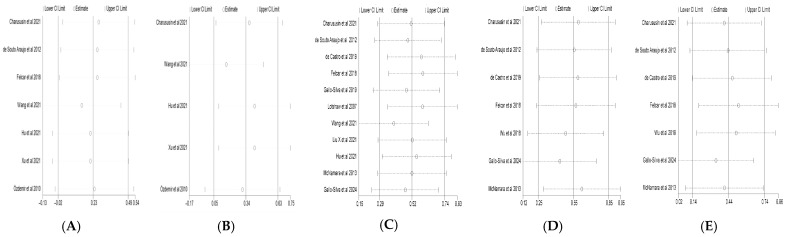

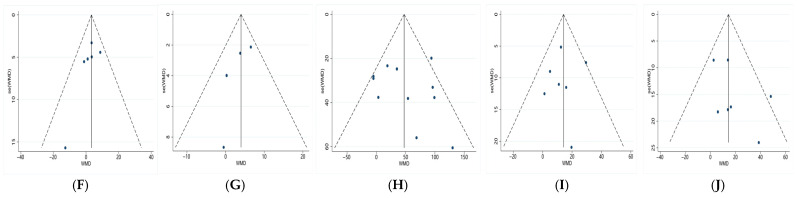
Sensitivity analyses and publication bias assessments: (**A**–**E**) sensitivity analyses for FEV_1_% predicted, FEV_1_/FVC, 6MWD, MIP, and MEP, respectively; (**F**–**J**) funnel plots for FEV_1_% predicted, FEV_1_/FVC, 6MWD, MIP, and MEP, respectively. (**A**) [15,23,24,26,29,30,32]; (**B**) [23,26,29,30,32]; (**C**) [10,15,23,24,27,28,30,32,33,34]; (**D**) [10,15,23,24,25,27,33]; (**E**) [10,15,23,24,25,27,33].

Sensitivity analyses were performed for all primary outcomes using a leave-one-out approach, whereby each study was sequentially removed from the meta-analysis (Figure 7A–E). The results showed that, after exclusion of any single study, the pooled effect sizes for all outcomes fluctuated within a limited range. The direction of the effects remained consistent with the main analyses, and the 95% confidence intervals largely overlapped with those of the primary pooled estimates.

No individual study was found to exert a decisive influence on the overall pooled effects, indicating that the results of the present meta-analysis are robust.

### 3.6. Publication Bias

Publication bias was assessed using funnel plots together with Begg’s rank correlation test and Egger’s regression test (Figure 7F–J). Visual inspection indicated that the funnel plots for all outcomes were generally symmetrical, with no obvious asymmetry or small-study effects.

For FEV_1_% predicted, Begg’s test showed no significant publication bias (Z = −1.80, *p* = 0.072; continuity-corrected Z = 1.65, *p* = 0.099), and Egger’s regression test revealed a non-significant intercept (bias = −1.21, t = −1.63, *p* = 0.164). Similarly, no significant publication bias was detected for FEV_1_/FVC by either Begg’s test (Z = −0.98, *p* = 0.327; continuity-corrected *p* = 0.462) or Egger’s test (*p* = 0.106).

For 6MWD, neither Begg’s test (Z = 1.17, *p* = 0.243; continuity-corrected Z = 1.09, *p* = 0.276) nor Egger’s regression test (bias = 3.06, t = 1.32, *p* = 0.219) indicated significant publication bias. Likewise, no evidence of publication bias was observed for MIP (Begg’s test: Z = 0.45, *p* = 0.652; Egger’s test: *p* = 0.814) or MEP (Begg’s test: Z = 0.45, *p* = 0.652; Egger’s test: t = 1.36, *p* = 0.231).

Overall, these results suggest that the findings of the present meta-analysis are unlikely to be substantially affected by publication bias.

## 4. Discussion

This systematic review and meta-analysis comprehensively compared the effects of water-based and land-based exercise on lung function, exercise capacity, and respiratory muscle function in patients with COPD. Overall, the findings indicate that, compared with conventional land-based exercise, water-based exercise demonstrates comparable or superior effects across multiple functional outcomes. In particular, consistent advantages were observed in improvements in exercise capacity and respiratory muscle function. These results provide new evidence supporting the role of water-based exercise as an effective intervention within pulmonary rehabilitation programs for patients with COPD.

### 4.1. Effects of Water-Based Exercise on Lung Function in Patients with COPD

First, the present study demonstrated that, compared with land-based exercise, water-based exercise significantly improved FEV_1_% predicted and FEV_1_/FVC in patients with COPD, with low between-study heterogeneity. These findings suggest that the aquatic environment may provide physiological stimuli to the respiratory system that differ from those elicited by conventional land-based training.

Although the observed improvements in FEV_1_% predicted and FEV_1_/FVC were statistically significant, their magnitude was modest and should be interpreted cautiously. In patients with COPD, spirometric indices are generally considered relatively insensitive to exercise-based interventions, and there is currently no universally accepted minimal clinically important difference (MCID) for FEV_1_% predicted or FEV_1_/FVC in this context [4,6]. Therefore, these changes should not be interpreted as reflecting substantial structural reversal of airflow limitation. Rather, the modest but consistent improvements observed in the present study may indicate enhanced ventilatory efficiency or respiratory mechanics and should be viewed as complementary to the more pronounced and clinically meaningful improvements observed in exercise capacity and respiratory muscle function [5,6,16].

From a mechanistic perspective, the effects of water-based exercise on lung function may be largely attributable to the sustained external pressure exerted by hydrostatic forces on the thorax and abdomen during water immersion [14]. Previous respiratory physiology studies have shown that increasing immersion depth markedly elevates external respiratory loading, requiring both inspiration and expiration to overcome additional resistance. This results in increased work of breathing and reduced chest wall compliance [37]. Further evidence indicates that such external loading enhances activation of the diaphragm and accessory respiratory muscles [38]. In patients with COPD, this hydrostatic pressure-induced increase in respiratory load may provide continuous and relatively mild training stimuli to the respiratory muscles without substantially increasing overall exercise intensity. Improvements in respiratory muscle function may, in turn, exert indirect beneficial effects on lung function.

Alterations in breathing patterns during water immersion or water-based exercise may also contribute to the observed improvements in lung function. Previous studies have reported that water immersion or water-based exercise can induce reductions in respiratory rate accompanied by increases in tidal volume or adjustments in breathing pattern, thereby improving ventilatory efficiency and reducing the proportion of dead space ventilation [39,40]. In patients with COPD, such breathing pattern modulation may help optimize ventilatory mechanics and partially alleviate manifestations of airway obstruction, which could be reflected in improvements in FEV_1_% predicted and FEV_1_/FVC [23]. It should be emphasized that these improvements are more likely to reflect enhanced ventilatory efficiency and respiratory control rather than a reversal of underlying airway structural abnormalities.

In addition, compared with land-based environments, water-based exercise is typically performed under conditions of higher ambient humidity and relatively stable temperatures, resulting in inhaled air that is closer to a warmed and humidified state. Previous respiratory physiology studies have suggested that warm and humidified inhaled air may help maintain airway mucosal hydration, improve mucociliary clearance, and reduce airway irritation and resistance [41,42,43]. For patients with COPD, particularly under conditions of increased ventilatory demand during exercise, these environmental differences may partially reduce airway burden and serve as an auxiliary mechanism contributing to improvements in lung function indices. However, direct evidence supporting an independent effect of aquatic environmental air conditions on lung function improvement remains limited, and the specific mechanisms and clinical relevance warrant further investigation.

### 4.2. Effects of Water-Based Exercise on Exercise Capacity in Patients with COPD

Reduced exercise capacity is a central manifestation of functional limitation and impaired quality of life in patients with COPD and represents a primary target outcome of pulmonary rehabilitation interventions [6]. The present meta-analysis demonstrated that, compared with land-based exercise, water-based exercise conferred a significant advantage in improving the six-minute walk distance (6MWD), with the magnitude of improvement reaching clinically relevant levels. This finding is consistent with results from several individual randomized controlled trials and further supports the potential benefits of water-based exercise in enhancing overall exercise capacity in patients with COPD [10,15].

From a mechanistic perspective, the beneficial effects of water-based exercise on exercise capacity may be primarily attributed to the buoyancy properties of water [44]. Biomechanical studies have demonstrated that body-weight bearing is substantially reduced during water immersion and decreases progressively with increasing immersion depth, thereby markedly lowering mechanical loading on the lower-limb joints and skeletal muscles [12,45]. This unloading effect enables individuals with functional limitations to perform exercise for longer durations or achieve higher total training volumes under conditions of reduced joint stress [46,47]. Accordingly, for patients with COPD who frequently present with lower-limb muscle weakness, joint degeneration, or fear of movement, buoyancy-induced mechanical unloading may be a prerequisite for maintaining sustained participation in exercise training.

In addition, water-based exercise may improve exercise capacity by reducing exercise-related dyspnea and enhancing patients’ subjective tolerance to training [48]. Several randomized controlled trials included in the present analysis consistently reported lower subjective dyspnea ratings, as assessed by the Borg scale, during aquatic training compared with land-based exercise [10,15]. In patients with COPD, exercise limitation is often driven primarily by dyspnea rather than peripheral muscle fatigue, and perceived breathlessness is considered a key determinant of exercise termination and intensity regulation [49]. Therefore, alleviation of dyspnea may directly influence behavioral tolerance to exercise, allowing patients to sustain prescribed intensities or prolong the duration of individual training sessions [49]. Consequently, patients may complete longer training durations at comparable or even higher relative intensities, thereby increasing the effective training dose achieved [6,50]. Such increases in training dose driven by improved subjective tolerance may represent one of the key mechanisms underlying the pronounced improvements in 6MWD observed with water-based exercise.

Notably, subgroup analyses in the present study indicated that improvements in 6MWD associated with water-based exercise were more pronounced during short-term interventions (≤8 weeks). Previous pulmonary rehabilitation research has similarly shown that functional outcome improvements are often most evident during the early phases of intervention, followed by a gradual plateau [6,51]. These early gains are thought to primarily reflect reversal of deconditioning, optimization of movement strategies, and neurobehavioral adaptations, rather than linear and sustained structural enhancements, resulting in a deceleration of improvement rates over time [16,51,52].

Furthermore, as a composite functional outcome, 6MWD is subject to physiological ceiling effects. As patients approach their current functional limits, further improvements require disproportionately greater training stimuli and longer intervention durations [16,51]. At the same time, with prolonged intervention periods, patients in land-based exercise groups may also accrue training-induced adaptations, thereby partially narrowing the performance gap between the two exercise modalities [15,53].

Taken together, the observed short-term superiority of water-based exercise may primarily reflect its advantages in enhancing early exercise tolerance and training adherence. However, the extent to which intervention duration modulates these effects over the long term remains to be clarified through future studies with larger sample sizes and extended follow-up periods.

### 4.3. Effects of Water-Based Exercise on Respiratory Muscle Function

Respiratory muscle dysfunction constitutes a key pathological basis of dyspnea and exercise limitation in patients with COPD and represents a clinically meaningful functional outcome in pulmonary rehabilitation [54,55,56]. The findings of the present study showed that, compared with land-based exercise, water-based exercise significantly improved both maximal inspiratory pressure (MIP) and maximal expiratory pressure (MEP) in patients with COPD, with low between-study heterogeneity. These results indicate a relatively consistent and reliable effect of water-based exercise on respiratory muscle function across different study settings.

The aquatic environment may offer distinct advantages in providing training stimuli to the respiratory muscles. Hydrostatic pressure acts continuously on the thorax and abdomen throughout the entire respiratory cycle. During inspiration, it increases the external resistance that must be overcome for chest wall expansion, while during expiration, abdominal compression elevates expiratory load. Consequently, both inspiratory and expiratory muscles are engaged in resistive regulation throughout the breathing cycle [57,58,59]. This bidirectional loading characteristic distinguishes water-based exercise from most forms of land-based aerobic training and provides a more direct and sustained stimulus to the respiratory musculature.

Previous studies further support these mechanistic considerations. Investigations into the respiratory physiological responses to water-based exercise have demonstrated significant increases in neural respiratory drive and respiratory muscle work, reflecting an overall elevation in respiratory muscle loading during water-based activity [60,61]. In patients with COPD, such naturally imposed loading derived from the aquatic environment may facilitate adaptive increases in respiratory muscle strength without the need to introduce additional or complex training modalities [27].

Compared with conventional land-based aerobic exercise, water-based exercise does not require specialized respiratory muscle training equipment to provide sustained and bidirectional resistive loading to the respiratory muscles [57,58]. This integrated loading pattern differs from targeted inspiratory muscle training (IMT), which primarily focuses on inspiratory muscles, and may therefore be more conducive to simultaneous improvements in both MIP and MEP [62]. Previous systematic reviews on specific respiratory muscle training have confirmed that increasing respiratory muscle load can significantly enhance respiratory muscle strength and related functional outcomes [63]. The present findings suggest that water-based exercise may, to some extent, elicit comparable training effects through a more integrated and physiologically natural mode of stimulation.

In addition to overall intervention effects, the water-based exercise protocols included in this review varied in key characteristics, including immersion depth, water temperature, and exercise modality, which may contribute to differences in observed effect sizes.

Among these factors, immersion depth is likely to be particularly influential because of its direct relationship with hydrostatic pressure. Increasing immersion depth increases external pressure on the thorax and abdomen, thereby elevating respiratory loading and activation of inspiratory and expiratory muscles. Such loading may provide a continuous, low-intensity training stimulus to the respiratory muscles, potentially contributing to improvements in respiratory muscle function and exercise capacity observed with water-based interventions [12,14].

In contrast, the potential influence of water temperature and exercise modality could not be meaningfully compared, as these parameters were inconsistently reported and often combined across studies. Therefore, any comparison across different aquatic conditions in the present review should be regarded as exploratory and qualitative rather than definitive. These observations underscore the need for standardized reporting of key aquatic parameters in future trials to facilitate clearer interpretation of dose–response relationships in water-based pulmonary rehabilitation.

## 5. Conclusions

This systematic review and meta-analysis comprehensively compared the effects of water-based and land-based exercise on lung function, exercise capacity, and respiratory muscle function in patients with chronic obstructive pulmonary disease (COPD). The findings indicate that, compared with conventional land-based exercise, water-based exercise demonstrates consistent advantages in improving key functional outcomes, particularly exercise capacity and respiratory muscle function, while also yielding modest improvements in lung function indices.

Based on the available evidence, water-based exercise should not be regarded merely as an equivalent alternative to land-based exercise, but rather as an intervention with potential advantages in specific functional domains. The unique properties of the aquatic environment may enhance exercise tolerance and provide sustained loading to the respiratory muscles, thereby facilitating functional improvements. As an integral component of pulmonary rehabilitation, water-based exercise may serve as a valuable adjunct to traditional land-based training under appropriate conditions. In particular, for patients with COPD who present with lower-limb muscle weakness, joint degeneration, obesity, or poor tolerance to land-based exercise, water-based exercise may help achieve comparable or even superior functional benefits while maintaining a favorable safety profile.

Despite the comprehensive synthesis of available randomized controlled evidence provided by the present systematic review and meta-analysis, several limitations should be acknowledged. First, the overall sample sizes of the included studies were relatively small, and substantial heterogeneity existed in intervention protocols, including exercise modality, training intensity, and intervention duration. In addition, follow-up periods were generally short, which limited the evaluation of sustained or long-term clinical outcomes. Second, blinding of participants and personnel is inherently challenging in exercise-based interventions, which may have introduced performance bias despite appropriate randomization procedures. Third, although variability in water-based exercise conditions was recognized as a potential modifier of intervention effects, reporting of key aquatic parameters was limited and inconsistent. Specifically, only a small number of studies reported water temperature, and fewer than half reported immersion depth or level, precluding quantitative exploration of these factors through subgroup analysis or meta-regression. As a result, the potential influence of specific aquatic conditions on effect sizes could only be discussed qualitatively rather than examined statistically.

In light of these limitations, future research should prioritize large-scale, methodologically rigorous randomized controlled trials with longer follow-up durations. Greater standardization and transparent reporting of key water-based exercise parameters—including exercise intensity, water temperature, immersion depth, and training frequency—would enhance comparability across studies and enable more robust evaluation of dose–response relationships. Furthermore, beyond traditional functional outcomes, future studies should incorporate clinically relevant endpoints such as exacerbation frequency, hospitalization, health-related quality of life, and long-term prognosis to more comprehensively assess the clinical value of water-based exercise within integrated COPD management.

## Figures and Tables

**Table 1:** Database Search Strategies.

Database	Search Method	Search Strategy (Keywords/Subject Terms)
Pubmed	MeSH terms + free-text terms	(“Pulmonary Disease, Chronic Obstructive” [MeSH] OR “Chronic Obstructive Pulmonary Disease” OR COPD) AND (“Aquatic Exercise” [MeSH] OR “Water-based exercise” OR “Water exercise” OR “Aquatic training” OR “Hydrotherapy” OR “Aquatic rehabilitation”)
Web of science	Topic terms + free-text terms	TS = ((“Chronic obstructive pulmonary disease” OR COPD) AND (“Aquatic exercise” OR “Water-based exercise” OR “Water exercise” OR “Aquatic training” OR Hydrotherapy OR “Aquatic rehabilitation”))
Cochrane library	Title + Abstract + Keywords	(“Chronic obstructive pulmonary disease” OR COPD) AND (“Aquatic exercise” OR “Water-based exercise” OR “Water exercise” OR “Aquatic training” OR Hydrotherapy OR “Aquatic rehabilitation”)
Embase	Emtree + free-text	(‘Chronic obstructive lung disease’/exp OR ‘Chronic obstructive pulmonary disease’ OR COPD) AND (‘Aquatic exercise’/exp OR ‘Water based exercise’ OR ‘Water exercise’ OR ‘aquatic training’ OR hydrotherapy OR ‘Aquatic rehabilitation’)
CNKI	Subject terms + free-text terms	(“Chronic obstructive pulmonary disease” OR “COPD”) AND (“Aquatic exercise” OR “Water-based exercise” OR “Water exercise” OR “Hydrotherapy” OR “Aquatic rehabilitation” OR “Water-based traditional exercise”)
Wanfang and Vip	Subject terms + free-text terms	(“Chronic obstructive pulmonary disease” OR COPD) AND (“Aquatic exercise” OR “Hydrotherapy” OR “Aquatic rehabilitation”)

**Table 2:** Characteristics of the Included Studies.

Study/Year	Country/Region	Sample Size	Sex/Age (Year)	Disease Severity	Intervention Details	Frequency	Duration	Temperature and Depth	Outcomes
Charususin et al., 2021 [23]	Thailand	WBE: *n* = 7; LBE: *n* = 7	NR; WBE: 66 ± 8.1; LBE: 69 ± 4.9	WBE: GOLD II-III; LBE: GOLD II-III	WBE: water-based aerobic exercise + functional training; LBE: land-based aerobic + functional exercise	2/week	8 weeks	NR	FEV_1_%pre, FEV_1_/FVC, 6MWD, MIP, MEP
Felcar et al., 2018 [24]	Brazil	WBE: *n* = 20; LBE: *n* = 16	NR; WBE: 69 ± 9; LBE: 68 ± 8	WBE: FEV_1_%pred = 48(17), moderate to severe COPD; LBE: FEV_1_%pred = 46(14), moderate to severe COPD	WBE: high-intensity water-based endurance exercise + resistance exercise; LBE: high-intensity land-based endurance + resistance exercise	3/week	24 weeks	NR	FEV_1_%pre, 6MWD, MIP, MEP
Wu et al., 2018 [25]	China	WBE: *n* = 14; LBE: *n* = 15	NR; WBE:65 ± 11; LBE:65 ± 8	WBE: GOLD II-III; LBE: GOLD II-III	WBE: water-based mind–body exercise; LBE: land-based Mind–body exercise	2/week	6 weeks	temperature: 32 ± 2 °C; depth: xiphoid to clavicle level	MIP, MEP
Özdemir et al., 2010 [26]	Turkey	WBE: *n* = 25; LBE: *n* = 25	NR; WBE: 60.9 ± 8.8; LBE: 64.1 ± 8.9	WBE: FEV_1_ = 1.43 ± 0.52, LBE: FEV_1_ = 1.48 ± 0.49,	WBE: water-based aerobic exercise + breathing exercise; LBE: land-based aerobic + breathing exercise	5/week	4 weeks	NR	FEV_1_%pre, FEV1/FVC
Gallo-Silva et al., 2024 [27]	Brazil	WBE: *n* = 11; LBE: *n* = 11	NR; WBE: 65.5 ± 6.3; LBE: 66.3 ± 10.2	WBE: FEV_1_ = 1.6 ± 0.4; LBE: FEV_1_ = 1.7 ± 0.5	WBE: water-based aerobic exercise; LBE: land-based aerobic exercise	3/week	8 weeks	NR	6MWD, MIP, MEP
Lotshaw et al., 2007 [28]	America	WBE: *n* = 20; LBE: *n* = 20	NR; WBE: 65 ± 14; LBE: 71 ± 7	WBE: FEV_1_%pred = 47.1 ± 17; LBE: FEV_1_%pred = 44.4 ± 15.8	WBE: water-based interval aerobic exercise; LBE: land-based interval aerobic exercise	3/week	6 weeks	NR	6MWD
Xu et al., 2021 [29]	China	WBE: *n* = 18; LBE: *n* = 18	NR; WBE: 65–75; LBE: 65–75	WBE: FEV_1_%pred = 30–70%, moderate to severe COPD; LBE: FEV_1_%pred = 30–70%, moderate to severe COPD	WBE: water-based mind-body exercise; LBE: land-based Mind–body exercise	2/week	24 weeks	temperature: NR; depth: xiphoid to clavicle	FEV_1_%pre, FEV_1_/FVC
Hu et al., 2021 [30]	China	WBE: *n* = 18; LBE: *n* = 18	NR; WBE: 60–75; LBE: 60–75	WBE: FEV_1_%pred = 30–70%, moderate to severe COPD; LBE: FEV_1_%pred = 30–70%	WBE: water-based mind–body exercise; LBE: land-based mind-body exercise	3/week	24 weeks	temperature: NR; depth: xiphoid to clavicle	FEV_1_%pre, FEV_1_/FVC, 6MWD
Liu X et al., 2021 [31]	China	WBE: *n* = 16; LBE: *n* = 17	NR; WBE: 65 ± 11; LBE: 65 ± 8	WBE: moderate COPD; LBE: moderate COPD	WBE: water-based mind–body exercise; LBE: land-based mind–body exercise	2/week	12 weeks	temperature: NR; depth: sternal to clavicle	6MWD
Wang et al., 2021 [32]	China	WBE: *n* = 25; LBE: *n* = 25	Male/Female; WBE: 60.3 ± 3; LBE: 63.1 ± 1.53	WBE: GOLD II-III; LBE: GOLD II-III	WBE: water-based aerobic exercise + respiratory muscle exercise; LBE: land-based aerobic respiratory muscle exercise	5/week	8 weeks	temperature: 30–34 °C; depth: 1–1.4 m	FEV_1_%,pre FEV_1_/FVC, 6MWD
de Souto Araujo et al., 2012 [15]	Brazil	WBE: *n* = 8; LBE: *n* = 13	NR; WBE: 62.4 ± 9.9; LBE: 56.9 ± 7.9	WBE: moderate to severe COPD; LBE: moderate to severe COPD	WBE: water-based aerobic exercise + resistance exercise; LBE: land-based aerobic + resistance exercise	3/week	8 weeks	NR	FEV_1_%pre, 6MWD, MIP, MEP
de Castro et al., 2019 [33]	Brazil	WBE: *n* = 14; LBE: *n* = 17	NR; WBE: 65 ± 8; LBE: 64 ± 8	WBE: FEV_1_%pred = 48%, moderate to severe COPD; LBE: FEV_1_%pred = 51%, moderate to severe COPD	WBE: high-intensity water-based endurance exercise + strength exercise; LBE: high-intensity land-based endurance + strength exercise	3/week	12 weeks	NR	6MWD, MIP, MEP
Gallo-Silva et al., 2019 [34]	Brazil	WBE: *n* = 10; LBE: *n* = 9	NR; WBE: 66.3 ± 6.5; LBE: 66.5 ± 9.5	WBE: FEV_1_ = 1.5–1.7, moderate COPD; LBE: FEV_1_ = 1.6–1.8, moderate COPD	WBE: water-based interval aerobic exercise; LBE: land-based interval aerobic exercise	3/week	8 weeks	NR	6MWD
McNamara et al., 2013 [10]	Australia	WBE: *n* = 15; LBE: *n* = 15	NR; WBE: 72 ± 9; LBE: 72 ± 9	WBE: FEV_1_%pred = 59 ± 15, moderate COPD; LBE: FEV_1_%pred = 60 ± 14, moderate COPD	WBE: water-based aerobic exercise; LBE: land-based aerobic exercise	3/week	8 weeks	NR	6MWD, MIP, MEP

Abbreviations: WBE, water-based exercise; LBE, land-based exercise; NR, not reported.

**Table 3:** Risk of Bias Assessment for Included Studies.

Studies	Randomization	Deviations	Missing Data	Outcome Measurement	Selective Reporting	Overall Bias
Charususin et al., 2021 [23]	Low risk	Low risk	Moderate	Low risk	Low risk	Moderate
Felcar et al., 2018 [24]	Low risk	Low risk	Low risk	Low risk	Low risk	Low risk
Wu et al., 2018 [25]	Low risk	Low risk	Low risk	Low risk	Low risk	Low risk
Özdemir et al., 2010 [26]	Low risk	Low risk	Moderate	Low risk	Low risk	Moderate
Gallo-Silva et al., 2024 [27]	Low risk	Low risk	Low risk	Low risk	Low risk	Low risk
Lotshaw et al., 2007 [28]	Moderate	Low risk	Moderate	Low risk	Moderate	Moderate
Xu et al., 2021 [29]	Low risk	Low risk	Low risk	Low risk	Low risk	Low risk
Hu et al., 2021 [30]	Low risk	Low risk	Low risk	Low risk	Low risk	Low risk
Liu X et al., 2021 [31]	Moderate	Moderate	Low risk	Low risk	Low risk	Moderate
Wang et al., 2021 [32]	Low risk	Moderate	Moderate	Low risk	Moderate	Moderate
de Souto Araujo et al., 2012 [15]	Low risk	Low risk	Low risk	Low risk	Low risk	Low risk
de Castro et al., 2019 [33]	Low risk	Moderate	Moderate	Low risk	Low risk	Moderate
Gallo-Silva et al., 2019 [34]	Moderate	Low risk	Moderate	Low risk	Low risk	Moderate
McNamara et al., 2013 [10]	Low risk	Low risk	Low risk	Low risk	Low risk	Low risk

## Data Availability

The original contributions presented in this study are included in the article. Further inquiries can be directed to the corresponding author.
